# Venomous Bites

**Published:** 1867-08

**Authors:** James T. Newman

**Affiliations:** Chicago


### VENOMOUS BITES.

By JAMES T. NEWMAN, M.D., Chicago.

Much has been said and written, of late years, on the subject which this article professes to treat. It has fallen to my lot to see several cases of such character:—three from the bite of the rattlesnake (*crotalus horridus*); one from the bite of the spider; one from the sting of the scorpion.

On the 10th of August, 1866, I was called to see a man who had been bitten on the right tendo Achillis. He had been bitten about three hours before I saw him. I found him in the most excruciating agonies imaginable. His whole right side was swelled, all the way from the bitten part up to the shoulder; his pulse was very rapid, and varied from 130 to 145; respiration also varied, from 36 to 40; the whole frame was in a high state of nervous trepidation; he also foamed at the mouth, gnashed his teeth, and violent spasms would ensue. I was four miles from town, without a particle of medicine in my possession. I ordered thirty grains of sul. morph. and a gallon of the best brandy. When it came, I took the morphia and divided it into six parts, and gave him one of them; in about ten minutes I gave him about eight ounces of brandy, and anxiously awaited the result. The swelling was rapidly covering the entire body. There I was, and not a physician within twelve miles of me. I was young and inexperienced, and had never seen a case of the kind I had to deal with; all that I knew was what I had read in the books, and my position was by no means enviable. At the end of an hour from the time the first dose was given, I repeated the morphia and brandy, and also applied a poultice of stramonium leaves. The spasms, I could now plainly see, were subsiding; this gave me hope. I then reduced the brandy to about four ounces every fifteen minutes. This treatment was kept up for six hours, changing the poultices every half hour. At the end of that time, he fell off into a sleep, which lasted four hours. The swelling now commenced to go down. When he awoke, I gave him more morphia in the same doses that I had commenced with. By this time, I had sent for four ounces aqua ammon.; two ounces of it I mixed with the same quantity of brandy. I had given him two doses of the ammonia and brandy, when he fell off into a sweet sleep, which lasted until 3 o'clock the next morning. I had then given him thirty grains of morphia, one gallon of brandy, and four ounces of ammonia. The swelling had almost subsided, except the part that had been bitten; it exuded a greenish fluid. I do not know whether this fluid was the virus or not; I ordered it to be wiped off. The result was, the patient recovered.

Case II. The recovery of the above-named case gained me the reputation of being able to cure the rattlesnake bite. I was sent for, on the 25th of August, of the same year, to attend a man who had been bitten by a rattlesnake. It was fourteen hours from the time he had received the wound when I saw him. He was swelled up nearly three times his former size; complained of having a sore throat; and would gulp when water or fluid were brought near him. I, however, opened his mouth and poured down a glass of brandy; he immediately vomited it up. I then gave him three grains of morphia, which was also ejected from the stomach. He soon sank into a state of coma and died.

Case III. A boy, about 10 years old, was sent by his mother to pick blackberries for tea, and was bitten on the hand by this terrible reptile. He threw down his cup and made for the house. I was living close by, and, hearing the screams of both mother and child, went to their relief. As soon as they told me what the matter was I took out my handkerchief, and, binding it around the little fellow's wrist, went to my office, which was about a quarter of a mile distant, and obtained some brandy, morphia, and ammonia. I then mixed the ammonia with the brandy, taking two drachms of ammonia and two of brandy, and gave it to him in about ten minutes; I also gave him a quarter of a grain of morphia, which was to be repeated every three hours; the ammonia and brandy was to be given every hour. The arm did not swell; the part that was wounded looked a little puffed, but it soon disappeared, and in a few days he was running around.

This treatment of morphia and brandy, for the bite of the *crotalus horridus*, was taken from a case that I had treated for the bite of the tarantula; the symptoms were exactly alike in the case of the snake bite and in that of the tarantula, with the exception that the pulse in the former was very rapid, and in the latter scarcely perceptible, all other symptoms were present.

Case IV. A child was bitten by that terrific insect, the tarantula. The parts immediately swelled up and spread over the whole body; circulation was impeded to a great extent; dimness and vertigo set in, with nausea and vomiting. I immediately gave it a fourth of a grain of morphia, in about ten minutes I also gave him two drachms of aqua ammon. and two of brandy, cauterized the parts with nitric acid, and kept up this treatment for two days, varying the doses as I thought proper. He recovered.

There is still another case which has fallen under my observation. This was poison produced by the sting of the scorpion. Pain, discoloration, and swelling were the immediate results. Ammonia externally applied, large doses of whiskey internally employed, and in a few hours the patient was relieved.

I will now take the opportunity to ask the Journal how hypodermic injections of the narcotic family would answer in such cases.

